# Machine learning-aided detection of heart failure (LVEF ≤ 49%) by using ballistocardiography and respiratory effort signals

**DOI:** 10.3389/fphys.2022.1068824

**Published:** 2023-01-19

**Authors:** Shen Feng, Xianda Wu, Andong Bao, Guanyang Lin, Pengtao Sun, Huan Cen, Sinan Chen, Yuexia Liu, Wenning He, Zhiqiang Pang, Han Zhang

**Affiliations:** ^1^ Department of Electronics and Information Engineering, South China Normal University (SCNU), Foshan, China; ^2^ School of Physics and Telecommunication Engineering, South China Normal University (SCNU), Guangzhou, China; ^3^ Department of Ultrasonography, The Second Affiliated Hospital of Guangzhou University of Chinese Medicine, Guangzhou, China; ^4^ Guangzhou SENVIV Technology Co., Ltd., Guangzhou, China

**Keywords:** heart failure, ballistocardiography, respiratory, classifier, home monitoring

## Abstract

**Purpose:** Under the influence of COVID-19 and the in-hospital cost, the in-home detection of cardiovascular disease with smart sensing devices is becoming more popular recently. In the presence of the qualified signals, ballistocardiography (BCG) can not only reflect the cardiac mechanical movements, but also detect the HF in a non-contact manner. However, for the potential HF patients, the additional quality assessment with ECG-aided requires more procedures and brings the inconvenience to their in-home HF diagnosis. To enable the HF detection in many real applications, we proposed a machine learning-aided scheme for the HF detection in this paper, where the BCG signals recorded from the force sensor were employed without the heartbeat location, and the respiratory effort signals separated from force sensors provided more HF features due to the connection between the heart and the lung systems. Finally, the effectiveness of the proposed HF detection scheme was verified in comparative experiments.

**Methods:** First, a piezoelectric sensor was used to record a signal sequences of the two-dimensional vital sign, which includes the BCG and the respiratory effort. Then, the linear and the non-linear features w.r.t. BCG and respiratory effort signals were extracted to serve the HF detection. Finally, the improved HF detection performance was verified through the LOO and the LOSO cross-validation settings with different machine learning classifiers.

**Results:** The proposed machine learning-aided scheme achieved the robust performance in the HF detection by using 4 different classifiers, and yielded an accuracy of 94.97% and 87.00% in the LOO and the LOSO experiments, respectively. In addition, experimental results demonstrated that the designed respiratory and cardiopulmonary features are beneficial to the HF detection (LVEF ≤49% ).

**Conclusion:** This study proposed a machine learning-aided HF diagnostic scheme. Experimental results demonstrated that the proposed scheme can fully exploit the relationship between the heart and the lung systems to potentially improve the in-home HF detection performance by using both the BCG, the respiratory and the cardiopulmonary-related features.

## 1 Introduction

Heart failure (HF) is a kind of clinical syndrome caused by the abnormal cardiac structure or function, which leads to ventricular filling or ejection dysfunction. As the send-stage manifestation of all cardiovascular diseases, chronic HF brings serious burdens including the poor prognosis and the high mortality to many patients’ families and our society (Hao et al., 2019). Also, as reporting in a global survey over 40 countries (Savarese et al., 2022), the reduced left ventricular ejection fraction (LVEF ≤49% ) resulted by the HF can bring significant mortality rate. Therefore, early detection of HF is essentially important recently, especially for elderly patients with HF (LVEF ≤49% ).

To better detect the body situation, some patients with hypertension and myocardial infarction and other heart diseases are suggested to perform regular medical checkups and early detection of HF in hospitals. For the dynamic management of chronic HF, regular review in hospitals to prevent the decompensation event is necessary (Society of Cardiology, 2018). Specifically, in the clinical diagnosis for the HF, echocardiography is regarded as one of the gold standards, and usually used to assess the systolic and diastolic capacity of the heart. In addition to the echocardiography, many techniques including X-rays, electrocardiogram (ECG) and brain natriuretic peptides (BNP) can be utilized as clinical key indicators to aid the HF diagnosis. However, the in-hospital HF diagnosis is challenging recently. On the one hand, under the influence of COVID-19, it may be inconvenient to the in-hospital routine check-ups related to the HF. On the other hand, many elderly patients are limited in the mobility, and unable to afford the high diagnostic cost for the inpatient check-ups. Therefore, it is worthy to develop the off-hospital/in-home HF detection scheme currently (Dickinson et al., 2018).

For the off-hospital/in-home HF detection, considering the benefits that the ballistocardiography (BCG) signals can conveniently monitor the body vibration caused by cardiac contraction and blood circulation, BCG is widely utilized for the in-home detection of various cardiac diseases recently (Wen et al., 2019). Specifically, Starr et al. first notified the clinical significance of BCG signals recorded by a mechanical bed, where the mathematical relationship between BCG and cardiac output (CO) was revealed (Starr et al., 1939), and the morphology difference between patients suffering from different types of cardio vascular (CV) diseases was analyzed (Starr and Schroeder, 1940), respectively. Also, the BCG signals were used to evaluate the cardiac contractility by the combined features with the ECG signals (Inan et al., 2009; Mozziyar et al., 2011; Ashouri et al., 2016). Since the correlation between the cardiac contractility and the heart functions, both the BCG and the ECG signals were applied to detect the HF decompensation (Giovangrandi et al., 2012; Etemadi et al., 2014; Aydemir et al., 2019). Similarly, Chang et al. calculated the waveform fluctuation metric at rest (WFMR) through the beat-to-beat features of the BCG signals to improve the quantitative analysis of the HF diseases (Chang et al., 2020). Generally, it was strongly demonstrated that the BCG per beat features can be effectively used for the HF detection in the above studies. However, considering the facts that many HF patients usually suffer from the weakened ventricular contractility, the mitral regurgitation and other symptoms, the resulted rhythm irregularity and the corresponding morphology diversity across different subjects bring a great challenge in the quality assessment of the BCG signals. Moreover, the respiratory effort signals, defined as the signals representing the energy consuming activity of the respiratory muscles (de Vries et al., 2018), and also is one of the important reference indicators for the clinical HF diagnosis (Siniorakis et al., 2018; Hamazaki et al., 2019), are rarely studied and developed in many existing works.

To overcome those above shortcomings, we proposed a non-contact sensing aided scheme for the in-home HF detection without quality assessment of BCG signals in this paper. Specifically, the piezoelectric sensing was used to acquire cardiopulmonary signs of various HF patients, where the signs contain the BCG and the respiratory effort information. Unlike the existing works, the proposed scheme performed the feature extractions from the BCG and the respiratory effort signals without the heartbeat location, where the extracted features finally identified the symptoms of the HF by using several typical classifiers. To validate the performance of the proposed HF detection scheme, the control experiments in terms of the leave-one-out (LOO) and the leave-one-subject-out (LOSO) rules were investigated. Experimental results demonstrated that the proposed scheme, in comparison with the existing studies, shows robust to HF (LVEF ≤ 49%) with low absolute global longitudinal strain (absolute GLS < 20%). Furthermore, the statistical analysis revealed that the respiratory and the cardiopulmonary features have the great diagnostic significance for the HF diseases.

## 2 Materials and methods

### 2.1 Experimental devices and protocol

**FIGURE 1:**
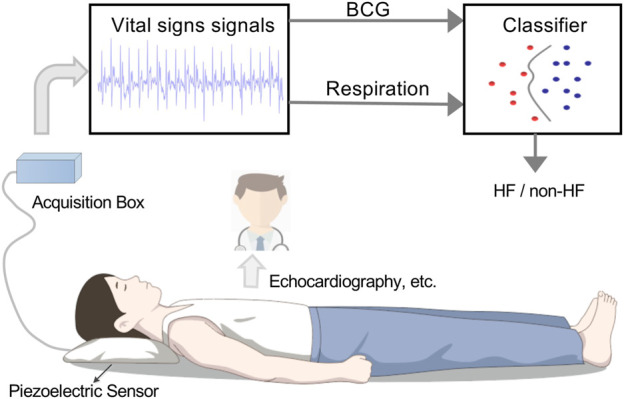
Heart failure detection by using non-contact recorded vital signs.

The overall experimental procedure is shown in Figure 1, the non-contact sensing device consists of a vital sign acquisition module and a signal processing module. The vital sign acquisition module was made by a piezoelectric sensor with a sampling rate of *f*_*s*_ = 1*k*Hz, and used to collect the vital signs under the head and neck. The aim of the signal processing module is to convert the collected signals as 12-bit analog-to-digital data. Furthermore, the echocardiography was recorded by EPIQ 7C (Philips).

Specifically, each subject was first asked to supine on an instrumented bed and keep soldier sleeping position for the 5 min baseline restoration and followed by vital sign signal acquisition. Before and during the 10 min period of the vital signs collection, all subjects did not have any exercise and kept the fixed positions. The recorded data are involved the non-contact sensing device and the echocardiography, where the collected vial signs contain the information of both the BCG and the respiratory effort from the head and necks of the subjects (Liu et al., 2021). After the data acquisition, some professional cardiologists made the HF diagnosis by the echocardiography signals, and other recorded data was selected by the above results. For a simpler interpretation, GLS was taken as absolute value in this study. Finally, the selected data from the non-contact sensing device was used in the following procedures of the proposed scheme.

### 2.2 Inclusion and exclusion criteria

To reduce the influence of other extraneous factors, the enrolled standards for the HF group are as follows.

• The HF diagnosis guidelines (Society of Cardiology, 2018).
• LVEF ≤49% and GLS <20% (Park et al., 2018).
• The diagnosed HF patients with complications of many heart diseases including the coronary artery disease, and the structural heart abnormalities resulted by the heart attack or the hypertension (all subjects received medications, and some of them has the coronary PCI or pacemakers.).

As a control group, the standards for the healthy group are as follows.

• Normal blood glucose, lipids, blood pressure, blood routine, liver function, and kidney function.
• Normal ECG (note: occasional atrial premature can be included as appropriate).
• No history of the medication affecting the cardiovascular system.
• No structural heart disease and normal cardiac function on the echocardiography (LVEF >50%), mild or less valvular regurgitation can be included. The criteria using the HFA-PEFF score is applied to classify the subjects with preserved LVEF (HFpEF) from all potential healthy candidates with LVEF >50% (Pieske et al., 2019).

### 2.3 Study population

**FIGURE 2:**
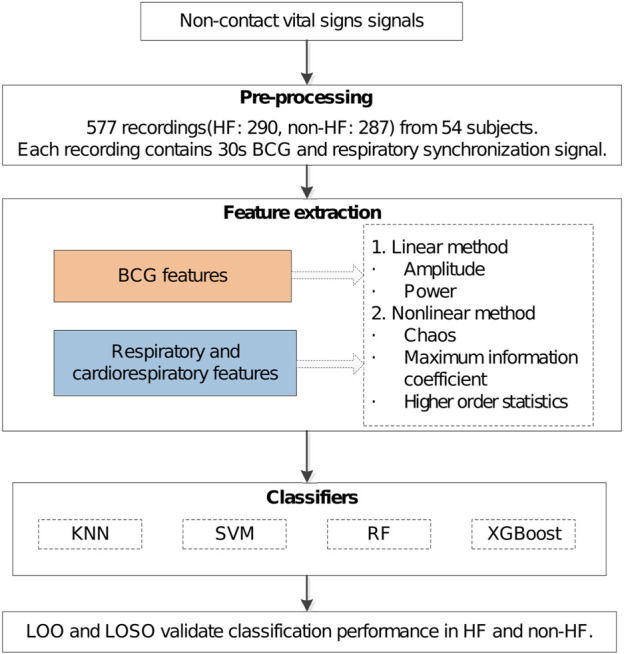
Overall diagram.

A total of 54 subjects in the age range of 23–92 years participated in the study, i.e., 24 healthy subjects and 30 HF (LVEF ≤49% ) patients. The details of the subjects are listed in Table 1. All subjects who participated in this study were recruited by the Second Affiliated Hospital of Guangzhou University of Chinese Medicine (Guangdong Hospital of Chinese Medicine) in Guangzhou, China, including the volunteers, the routine physical examiners, and the HF patients. The study protocol has been reviewed and approved by the Ethics Committee of Guangdong Provincial Hospital of Traditional Chinese Medicine (ZE 2022–123). All subjects obtained the informed consent before their participation in this study. The flowchart diagram of the proposed non-contact HF diagnosis method is shown in Figure 2.

**TABLE 1:** A description of the subjects. BMI: body mass index. LVEF: left ventricular ejection fraction. GLS: global longitudinal strain. Value performance by mean ± standard deviation.

		Gender							
	Number	(male	Age	Height	Weight	BMI	LVEF	GLS	Doppler
		/female)	(year)	(cm)	(kg)	(kg/m^2^)	(%)	(%)	ultrasonography
HF	30	20/10	61.80 ± 13.63	156.29 ± 8.24	61.63 ± 12.38	23.62 ± 4.02	38.99 ± 4.02	11.41 ± 3.76	Low, medium, and large volumes of regurgitation in 15, 6 and 9 cases, respectively
non-HF	24	19/5	45.79 ± 14.03	166.58 ± 8.65	67.29 ± 11.86	24.25 ± 3.29	69.21 ± 3.71	21.16 ± 2.40	No abnormal, small and moderate regurgitation in 9, 13 and 2 cases, respectively

### 2.4 Pre-processing

The key purpose of performing the pre-processing is to process and divide the collected data into several data sets for the HF detection, where the first step is associated with the signal separation, and the second one is related to the sample grouping.

**FIGURE 3:**
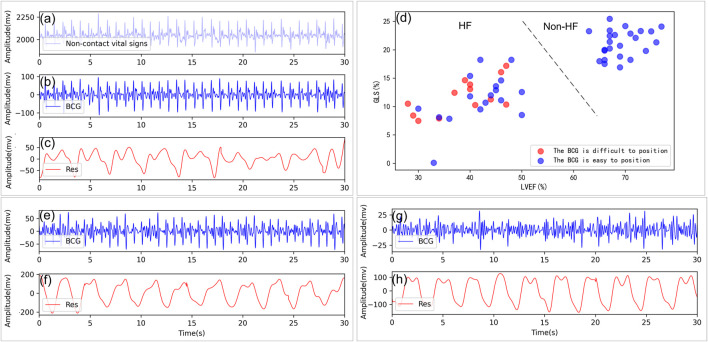
Non-contact vital sign signal processing and analysis. **(A)** Example of a non-contact vital sign signal collected. **(B**, **C)** Example of BCG and respiratory signals isolated from a healthy subject, GLS = 21.30%, LVEF = 76%. **(D)** Distribution of the J-peaks of the BCG for those who are difficult located (red circles) and those who are easier located (blue circles) in terms of LVEF, GLS. **(E**, **F)** Example of BCG and respiratory signals from a patient with HF with BCG can be located, GLS = 9.62%, LVEF = 30%. **(G**, **H)** Example of BCG and respiratory signals from a patient with HF with heartbeat are very difficult to locate, GLS = 16.07%, LVEF = 46%. Res: respiratory signal.

For the first step, similar to (Jingxian et al., 2020), the peak-to-mean ratio detection of the 1s time-scale signals was performed to remove the artifact interference and separate the BCG signals and the respirator effort contributions from the collected data. Specifically, the morphological and the low-pass filters were sequentially exploited to obtain the respiratory effort signals. Then we removed the separated respiratory effort signals from the collected signals and obtained the new vital sign sequences. Given such new sequences, a 4th-order Butterworth filter (2 Hz ∼8.5 Hz) was applied to get the improved BCG signals. The results of the above signal separation can be found in Figure 3, where Figure 3A shows the 30s sign signals acquired *via* the piezoelectric sensing, and Figures 3B, C are the de-noised BCG and the respiratory effort signals, respectively.

Similar to (Chang et al., 2020), the BCG and the respiratory effort signals were first divided into multiple consecutive epochs of 30s in a non-overlapped manner. As a result, the total number of the recordings was 577, where each recording included the 30s synchronously BCG and the respiratory effort epoch. In details, 290 recordings were associated with the HF patients while others are related to the non-HF subjects. Among them, recordings were evenly distributed from each subject to ensure no bias in the data and furthermore the universality of the experimental results. In the traditional studies, the HF diseases can be easy detected by the typical waveforms of the BCG signals (Carlson et al., 2020; Liu et al., 2021). However, the corresponding BCG signals are usually irregular in rhythm and morphology due to disordered cardiac movement (HF patients with LVEF ≤49% and GLS <20% ) (Siniorakis et al., 2018; Hamazaki et al., 2019; McDonagh et al., 2021). The irregular heartbeat waveforms of the BCG signals bring challenges for heartbeat location in the HF detection. By (Aydemir et al., 2019; Mai et al., 2022), we use signal-to-noise ratio (SNR) to access the signal quality of each BCG epoch. When SNR ≪ 4 dB, the whole (or part of) epoch of BCG signals are almost unable to identify. Figures 3B, C, E–H depicts the recorded BCG and the respiratory signals in terms of healthy subjects, HF patients with the heartbeat in BCG signals easily or difficult to be identified. It is noted that the heartbeat in BCG of 13 out of 30 HF patients was difficult to recognize due to the heart abnormality, as shown in Figure 3D, where the related existing methods are difficult to be applied (Aydemir et al., 2019; Chang et al., 2020). Therefore, to illustrate the robustness of our HF diagnostic method, we divided all 577 recordings into two groups in this experiment, i.e., Datasets 1 consists of 176 HF recordings (recognizable heartbeat in BCG) and 287 non-HF recordings, and Datasets 2 includes 114 HF recordings (unrecognizable heartbeat in BCG) and 287 non-HF recordings.

### 2.5 Feature extraction

#### 2.5.1 BCG features

Unlike the existing HF detection methods depended on the heartbeat location of the BCG signals in (Etemadi et al., 2014; Aydemir et al., 2019; Chang et al., 2020), our proposed scheme focused on the large scale features of the BCG signals, i.e., the 30 s epoch, where the hearbeat location was no longer needed. The corresponding features in the experiments were extracted in both linear and non-linear domains, as shown in Table 2.

**TABLE 2:** BCG features without localization assistance. sd: standard deviation. IQR: interquartile range.

Feature	Category	Name	Description
T1	Linear	*F*(*BCG*(*n*))	BCG amplitude coefficient
T2-T5		*Power*_*L*_ (*BCG*(*n*))) {*mean*, *sd*, *IQR*, *median*}	Power volatility of BCG on a fixed time scale
T6	Non-linear	*FE* (*BCG*(*n*))	BCG fuzzy entropy
T7-T10		*FE*_*L*_ (*BCG*(*n*)) {*mean*, *sd*, *IQR*, *median*}	Fuzzy entropy volatility of BCG on fixed time scale
T11		*LLE* (*BCG*(*n*))	BCG largest liapunov exponent
T12		*CD* (*BCG*(*n*))	BCG correlation dimension
T13-T16		*MIC*_*L*−1_(*BCG*(*n*)) {*mean*, *sd*, *IQR*, *median*}	MIC volatility of BCG on a fixed time scale
T17		*Skewness* (*BCG*(*n*))	BCG skewness
T18		*Kurtosis* (*BCG*(*n*))	BCG kurtosis

##### 2.5.1.1 Linear features

###### 2.5.1.1.1 Amplitude

The amplitude coefficient of the given signal *x*(*n*) can be expressed as

$$F\left(x\left(n\right)\right)=\frac{rms\left(x\left(n\right)\right)}{\max\left(x\left(n\right)\right)-\min\left(x\left(n\right)\right)}$$ (1)

where *rms* (⋅) is the root mean square function, max (⋅) indicates the maximum value function, and min (⋅) denotes the minimum value function. According to Eq. 1, we obtained the amplitude features of each BCG epoch by *F*(*BCG*(*n*)) in the experiments, where the notation *BCG*(*n*) was the separated BCG signals obtained from the above pre-progressing, and the amplitudes of the HF patient group were lower than that of the health subject group. This case is usually caused by that the HF patients suffer from the low myocardial contractility.

###### 2.5.1.1.2 Power

Inspired by the morphological difference between HF patients and healthy subjects (Chang et al., 2020), the volatility and the irregularity of the BCG singals can be measured and analyzed in the power-domain. To eliminate individualized differences, in our experiments, the signal was normalized using Z-score before the power calculation (Shi et al., 2022). As (Li et al., 2020), the signals *x*(*n*) was first divided into *L* segments *x*′ by the sliding window as

$$x_{L}^{\prime }=\left\{x_{1}^{\prime }\left(i\right),x_{2}^{\prime }\left(i\right),\ldots ,x_{L}^{\prime }\left(i\right),\;i=1,2,\ldots ,w*f_{s}\right\}$$ (2)

where $L=fix\left(\frac{N-w*f_{s}+t*f_{s}}{t*f_{s}}\right)$ represents the number of segments. *N* and *w* are the length of *x*(*n*) and the sliding window (the segment length), respectively. *t* indicates the time moving factor of the sliding window, *f*_*s*_ denotes the sampling rate, and *fix* (⋅) is the rounding function. The power of each segment was computed as

$$\begin{array}{ll} {Power}_{L}\left(x^{\prime }\right) & =\left\{\text{power}\left(x_{1}^{\prime }\left(i\right)\right),\text{power}\left(x_{2}^{\prime }\left(i\right)\right),\ldots ,\text{power}\left(x_{L}^{\prime }\left(i\right)\right),\;\right.\\ & \;\;\left.i,=,1,2,,,\ldots ,,,w,*,f_{s}\right\} \end{array}$$ (3)

where *power* (⋅) is the power function. As a result, the statistics *Power*_*L*_ (*BCG*(*n*)) were used to characterize the volatility of the BCG signals.

##### 2.5.1.2 Non-linear features

###### 2.5.1.2.1 Chaos

Chaos is defined as an uncertain or unpredictable random phenomenon presented by a deterministic system under many certain conditions, which can evaluate the level of the time-series signal disorder (Gupta et al., 2019). As a result, the chaotic features of the BCG time series, including fuzzy entropy (FE), largest Lyapunov exponent (LLE) and correlation dimension (CD), can be extracted to evaluate the disorder degree in BCG.

Firstly, FE (Chen et al., 2007) not only reflects the similarity between two vectors in the phase space, but also represents the complexity of the chaos system, defined as

$$FE\left(x\left(n\right)\right)=-\text{ln}\frac{C^{m+1}\left(r\right)}{C^{m}\left(r\right)}$$ (4)

where *m* is the embedding dimension, *r* indicates the similarity tolerance limit threshold, *C* ^*m*^(*r*) denotes the average of all fuzzy affiliations except itself. The FE of a BCG epoch was obtained by *FE* (*BCG*(*n*)) in this study with the setting *m* = 2, and *r* = 0.15. In addition, similar to the calculation for the power volatility, the FE of each BCG segment, i.e., *FE*_*L*_ (*BCG*(*n*)), was computed by using Eqs. 2, 4, which characterize the volatility of the FE series.

Next, we used two features including the LLE and the CD to better analyze the BCG signals (Procacia et al., 1983; Rosenstein et al., 1993), where LLE is the exponential rate of the convergence between two adjacent trajectories, and CD indicates the correlation between two phase points in the phase space. Their definition can be respectively expressed as

$$LLE\left(x\left(n\right)\right)=\frac{1}{\Delta t}<\text{ln}d_{\text{j}}\left(i\right)>$$ (5)

$$CD\left(x\left(n\right)\right)=\underset{r\to 0}{\text{lim}}\frac{\text{ln}C\left(r\right)}{\text{ln}\left(r\right)}$$ (6)

where <⋅> denotes the mean value, Δ*t* is the sampling interval, and *d*_j_(*i*) indicates the distance of the *j*-th pair of nearest neighbors after *i* discrete time steps. In the phase space, the function *C*(*r*) is the proportion between the number of the point pairs locating within the given radius *r* of the hyper-sphere and the number of all point pairs. Therefore, according to Eqs. 5, 6, we computed the LLE and the CD by the maximum value of the parameter embedding dimension *m* = 12 and the maximum value of the time delay factor *τ* = 40 ms as *LLE* (*BCG*(*n*)) and *CD* (*BCG*(*n*)), respectively.

###### 2.5.1.2.2 Maximum information coefficient

The maximum information coefficient (MIC) (Reshef et al., 2011) is a generalization of mutual information. In order words, the MIC can describe the association degree between two series (0: no correlation, 1: strong correlation) by a maximum information-based non-parametric exploration. The MIC between two random variables *x*_1_ and *x*_2_ can be expressed as

$$MIC\left(x_{1},x_{2}\right)=\underset{pq<B\left(s\right)}{\text{max}}\left\{M{\left(D\right)}_{p,q}\right\}$$ (7)

where *B*(*s*) = *s* ^0.6^ is a function of the sample number. *M*(*D*) is the mutual information on the grids p*q. Using Eqs. 2, 7, the MIC values of the two adjacent segments can be calculated to form an *L*−1 sequence, i.e., *MIC*_*L*−1_ (*x*(*n*)). As a result, we computed the MIC parameter of the BCG signals as *MIC*_*L*−1_(*BCG*(*n*)).

In the presence of the 4s sliding window and the 3s/2s/1s time moving factors, we calculated the mean, the standard deviation, and the interquartile range (IQR), and the median statistics for the above features, i.e., *Power*_*L*_ (*BCG*(*n*), *FE*_*L*_ (*BCG*(*n*)), and *MIC*_*L*−1_(*BCG*(*n*))), to serve the following experiments and analysis.

###### 2.5.1.2.3 Higher order statistics

Similar to (Bruser et al., 2012), the skewness and the kurtosis of the given signals *x*(*n*) can be respectively calculated as

$$Skewness\left(x\left(n\right)\right)=\frac{{\text{m}}_{3}\left(x\left(n\right)\right)}{{\text{m}}_{2}{\left(x\left(n\right)\right)}^{3/2}}$$ (8)

$$Kurtosis\left(x\left(n\right)\right)=\frac{{\text{m}}_{4}\left(x\left(n\right)\right)}{{\text{m}}_{2}{\left(x\left(n\right)\right)}^{2}}$$ (9)

where m_*k*_ (*x*(*n*)) denotes the *k*th sample moment around the mean of the signals *x*(*n*).

#### 2.5.2 Respiratory and cardiopulmonary features

Considering the facts that HF patients often suffer from the Respiratory aggravation symptoms, such as the chest tightness, the wheezing, the breath shortness, and the dyspnea due to the cardiac insufficiency (McDonagh et al., 2021), there are many connections between the respiration/cardiopulmonary-related features and the HF diseases. However, these features obtained from wearable devices lack attention recently and are rare to be applied into the HF detection. Motivated by this fact, in this study, we proposed to use these respiration-related features to improve the performance of the HF detection. These associated features can be found in Table 3. Similar to the above BCG-related features, many details of the respiration/cardiopulmonary-related feature calculation/extraction are presented as follows.

**TABLE 3:** Respiratory and cardiopulmonary features. sd: standard deviation. IQR: interquartile range.

Feature	Category	Name	Description
T19	Respiratory	*F*(*Res*(*n*))	Respiratory effort amplitude coefficient
T20		*FE* (*Res*(*n*))	Respiratory effort fuzzy entropy
T21-T24		*FE*_*L*_ (*Res*(*n*)) {*mean*, *sd*, *IQR*, *median*}	Fuzzy entropy volatility of respiratory signals on fixed time scales
T25		*Skewness* (*Res*(*n*))	Respiratory effort skewness
T26		*Kurtosis* (*Res*(*n*))	Respiratory effort kurtosis
T27	Cardiopulmonary	*F* ^*ratio*^ (*Res*(*n*), *BCG*(*n*))	Ratio of respiratory to BCG amplitude coefficient
T28		*Power* ^*ratio*^ (*Res*(*n*), *BCG*(*n*))	Ratio of respiratory to BCG signal power
T29-T32		$Power_{L}^{\mathrm{ratio}}\left(Res\left(n\right),BCG\left(n\right)\right)$	Volatility of respiration to BCG power ratio on a fixed time scale
		{*mean*, *sd*, *IQR*, *median*}	
T33		*FE* ^*sum*^ (*Res*(*n*), *BCG*(*n*))	Sum of respiratory and BCG signal fuzzy entropy

##### 2.5.2.1 Respiratory features

Considering that the breath shortness in HF patients leads to the enhanced respiratory effort, we chooosed the amplitude coefficient of the respiratory signals, i.e., *F*(*Res*(*n*)), to characterize the respiratory strength in the proposed scheme, The corresponding feature details can be found in Eq. 1.

Similarly, the FE of the respiratory-related epochs, *FE*(*Res*(*n*)), was calculated by Eqs. 2, 4 to characterize the volatility of the respiratory-related signals. Specifically, the corresponding window length and the time moving factor were set at 6s and 1s, respectively. As mentioned in the BCG-related feature extractions, many typical statistics including the skewness and the kurtosis related to the respiratory FE were calculated for the classifiers.

##### 2.5.2.2 Cardiopulmonary features

Similar to the respiratory effort, the connection between the heart and the lung systems can support that using the cardiopulmonary analysis to improve the heart detection performance. However, unlike the above feature extractions only relying on the BCG/respiratory signals, the cardiopulmonary features are associated with both the heart and the lung systems. As a result, the cardiopulmonary joint analysis can not only provide more benefits of reducing the effect caused by the occasional interference/noise in the BCG or the respiratory signals, but also eliminate the potential errors due to individual difference. The cardiopulmonary features, specifically, include the relative amplitude and power of respiratory effort and BCG, and quantify the overall complexity of these two kinds of signals.

According to the above considerations, the amplitude coefficient is defined as the ratio of the amplitude coefficients related to the respiratory and the BCG signals as

$$\begin{array}{ll} F^{\mathrm{ratio}}\left(Res\left(n\right),BCG\left(n\right)\right) & =\frac{F\left(Res\left(n\right)\right)}{F\left(BCG\left(n\right)\right)}\\ & =\frac{\frac{\text{rms}\left(Res\left(n\right)\right)}{\text{max}\left(Res\left(n\right)\right)-\text{min}\left(Res\left(n\right)\right)}}{\frac{\text{rms}\left(BCG\left(n\right)\right)}{\text{max}\left(BCG\left(n\right)\right)-\text{min}\left(BCG\left(n\right)\right)}} \end{array}$$ (10)

Similarly, the corresponding power features and the corresponding chaos features was respectively calculated as

$$Power^{\mathrm{ratio}}\left(Res\left(n\right),BCG\left(n\right)\right)=\frac{\sum_{n=1}^{N}{\left(Res\left(n\right)\right)}^{2}/N}{\sum_{n=1}^{N}{\left(BCG\left(n\right)\right)}^{2}/N}$$ (11)

$$FE^{\mathrm{sum}}\left(Res\left(n\right),BCG\left(n\right)\right)=FE\left(Res\left(n\right)\right)+FE\left(BCG\left(n\right)\right)$$ (12)

Usually, HF patients have shortness of breath and a reduced volume per beat, which results in the larger values of *F* ^*ratio*^ (*Res*(*n*), *BCG*(*n*)) and *Power* ^*ratio*^ (*Res*(*n*), *BCG*(*n*)) than that of the healthy candidates. Consequently, it is thus expected that the above cardiopulmonary features (10)–(12) can complement the individual differences possibly induced by single-channel features of BCG or respiratory signals. Also, we calculated the relative power volatility of respiration and BCG using Eqs. 2, 11, the recommended window and time moving factor are recommended to be adjusted as 2s. The statistics of $Power_{L}^{\mathrm{ratio}}\left(Res\left(n\right),BCG\left(n\right)\right)$ were calculated for the following classifiers in the experiments.

### 2.6 Classifiers

Based on the above extracted features related to the BCG/respiratory/cardiopulmonary signals, we applied four supervised classifiers to evaluate the performance of the HF detection. The classifiers include the K-Nearest Neighbor (KNN), the Support Vector Machine (SVM), the Random Forest (RF) and the eXtreme Gradient Boosting (XGBoost), where the features require to be normalized within [0,1] before performing the classification (Shi et al., 2022).

Among these four classifiers, the KNN (Ertuğrul and Tağluk, 2017) algorithm is the fastest algorithm, where the principle is to classify the new data points by those nearest *K* classified data points. However, the KNN has the limited performance in the complex classification boundary. To achieve better classification in the complex data space, SVM (Palaniappan et al., 2014) utilizes the sparsity between classified data points, i.e., only a few of points play important role to the classification boundary. Unlike the iterative processing of the SVM may cost much system resources, the RF (Sun et al., 2020) algorithm is based on the multiple decision tress without iterations. By the gradient boosting in the optimization theory, the XGBoost (Chen and Guestrin, 2016) classifier is a distributed enhancement with many benefits including the low complexity and the high flexibility.

### 2.7 Performance metrics

Similar to (Magrelli et al., 2021), the performance metrics include accuracy (Acc), sensitivity (Sen), specificity (Spe), F1 score (F1) and area under the curve (AUC), defined as

$$Acc=\frac{TP+TN}{TP+TN+FP+FN}$$ (13)

$$Sen=\frac{TP}{TP+FN}$$ (14)

$$Spe=\frac{TN}{TN+FP}$$ (15)

$$F1=\frac{2TP}{2TP+FP+FN}$$ (16)

where *TP* represents the number of correctly predicted positive samples, *TN* indicates the number of correctly predicted negative samples, *FP* is the number of negative samples predicted to be positive, and *FN* denotes the number of positive samples predicted to be negative. *AUC* is the area of receiver operating characteristic curve (ROC).

For the fairness in the evaluation, the LOO and the LOSO methods were used for the training and the testing phases, respectively. During the model training and testing phases, we also optimized each classifier by using the grid search method and obtained their highest classification accuracy.

## 3 Results

### 3.1 Performance of HF detection with different classifiers

**FIGURE 4:**
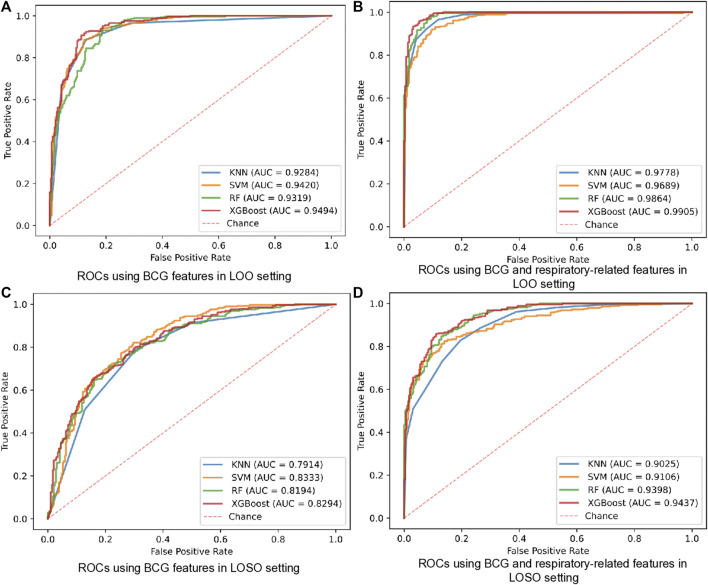
ROCs of HF diagnostics based on BCG and respiratory-related features using 4 classifiers in all datasets. **(A)** ROCs using BCG features in LOO setting. **(B)** ROCs using BCG and respiratory-related features in LOO setting. **(C)** ROCs using BCG features in LOSO setting. **(D)** ROCs using BCG and respiratory-related features in LOSO setting.

Firstly, we examined the performance of the proposed HF detection scheme in the presence of 4 different classifiers by the BCG, the respiratory and the cardiopulmonary-related features. Specifically, the performance metrics including the accuracy, the sensitivity, the specificity, the F1 score, and the AUC, are presented in Table 4 and Figure 4. These results showed that all 4 classifiers could provide the accuracy of over 91.16% under the LOO setting. Among them, the best performance was brought by the XGBoost classifier at 94.97% accuracy. In order to verify the stability of the performance, the LOSO cross-validation results were also given in Table 4 to provide person independent classification results. Under the LOSO setting showing the generalization ability of the proposed scheme, the best accuracy results of the proposed HF detection scheme were provided by the XGBoost. In addition, for all 4 classifiers, the performance brought by the BCG and respiration-related (respiratory and cardiopulmonary) features outperformed that of the BCG features under both the LOO and the LOSO setting. The above results indicated that the proposed scheme, which is based on the BCG, the respiratory and the cardiopulmonary features, significantly improved the detection performance of the HF diseases.

**TABLE 4:** LOO and LOSO classification results for HF diagnostics based on BCG and respiratory-related features using 4 classifiers. Resp: Respiratory and cardiopulmonary features.

Classifiers	Features	LOO					LOSO				
		Acc(%)	Sen(%)	Spe (%)	F1 (%)	AUC(%)	Acc(%)	Sen(%)	Spe (%)	F1 (%)	AUC(%)
KNN	BCG	87.88	88.62	87.11	88.01	92.84	74.18	78.97	69.34	75.45	79.14
	BCG & Resp	92.20	92.07	92.33	92.23	97.78	81.63	82.76	80.49	81.91	90.25
SVM	BCG	87.88	88.97	86.76	88.05	94.20	75.56	80.00	71.08	76.69	83.33
	BCG & Resp	91.16	92.07	90.24	91.28	96.89	83.36	82.76	83.97	83.33	91.06
RF	BCG	85.10	87.59	82.58	85.52	93.19	74.18	76.55	71.78	74.87	81.94
	BCG & Resp	93.93	94.83	93.03	94.02	98.64	85.44	86.55	84.32	85.67	93.98
XGBoost	BCG	89.43	90.00	88.85	89.54	94.94	74.18	74.48	73.86	74.35	82.94
	BCG & Resp	94.97	96.55	93.38	95.08	99.05	87.00	86.21	87.80	86.96	94.37

**FIGURE 5:**
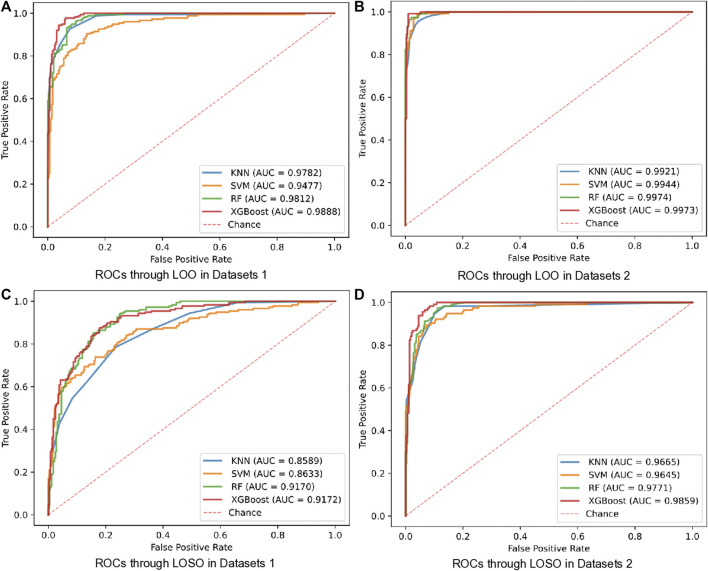
ROCs of LOO and LOSO for HF diagnostics over 2 datasets using 4 classifiers. **(A)** ROCs through LOO in Datasets 1. **(B)** ROCs through LOO in Datasets 2. **(C)** ROCs through LOSO in Datasets 1. **(D)** ROCs through LOSO in Datasets 2.

Comparing with that one of the main limitations in the existing BCG-based HF detection algorithm is the additional quality assessment, our proposed detection scheme can avoid this limitation by using more linear and non-linear features of both the heart and the lung systems. To verify the corresponding effectiveness, two types of dataset, i.e., the Datasets 1 (recognizable heartbeat in BCG) and the Datasets 2 (unrecognizable heartbeat in BCG) were used in performing our proposed HF detection method. The detailed experiment results are shown in Table 5 and Figure 5, which demonstrated that the Datasets 2 provided better classification performance than the Datasets 1 in all 4 classifiers under both the LOO and the LOSO settings. Among them, the best performance was brought by the XGBoost classifier at 99.00% and 94.01% under the LOO and the LOSO settings, respectively. All the above results showed that our proposed scheme addressed the limitation related to the heartbeat location in the existing BCG-based HF detection algorithms.

**TABLE 5:** LOO and LOSO classification results for HF diagnostics over 2 datasets using 4 classifiers. Datasets 1: HF patients of BCG easily localized and healthy subjects samples. Datasets 2: HF patients of BCG not easily localized and healthy subjects samples.

Classifiers	Datasets	LOO					LOSO				
		Acc(%)	Sen(%)	Spe(%)	F1(%)	AUC(%)	Acc(%)	Sen(%)	Spe(%)	F1(%)	AUC(%)
KNN	Datasets1	91.79	85.23	95.82	88.75	97.82	77.32	62.50	86.41	67.69	85.89
	Datasets2	95.76	93.86	96.52	92.64	99.21	91.02	81.58	94.77	83.78	96.65
SVM	Datasets1	88.55	81.82	92.68	84.46	94.77	79.05	71.02	83.97	72.05	86.33
	Datasets2	96.51	94.74	97.21	93.91	99.44	92.27	84.21	95.47	86.10	96.45
RF	Datasets1	90.71	86.36	93.38	87.61	98.12	82.94	72.16	89.55	76.28	91.70
	Datasets2	96.76	90.35	99.30	94.06	99.74	92.77	85.09	95.82	87.00	97.71
XGBoost	Datasets1	95.15	94.32	95.82	93.79	98.89	83.37	77.84	86.76	78.06	91.72
	Datasets2	99.00	99.12	98.95	98.26	99.73	94.01	86.84	96.86	89.19	98.59

### 3.2 Feature importance of LOO and LOSO experiments

**FIGURE 6:**
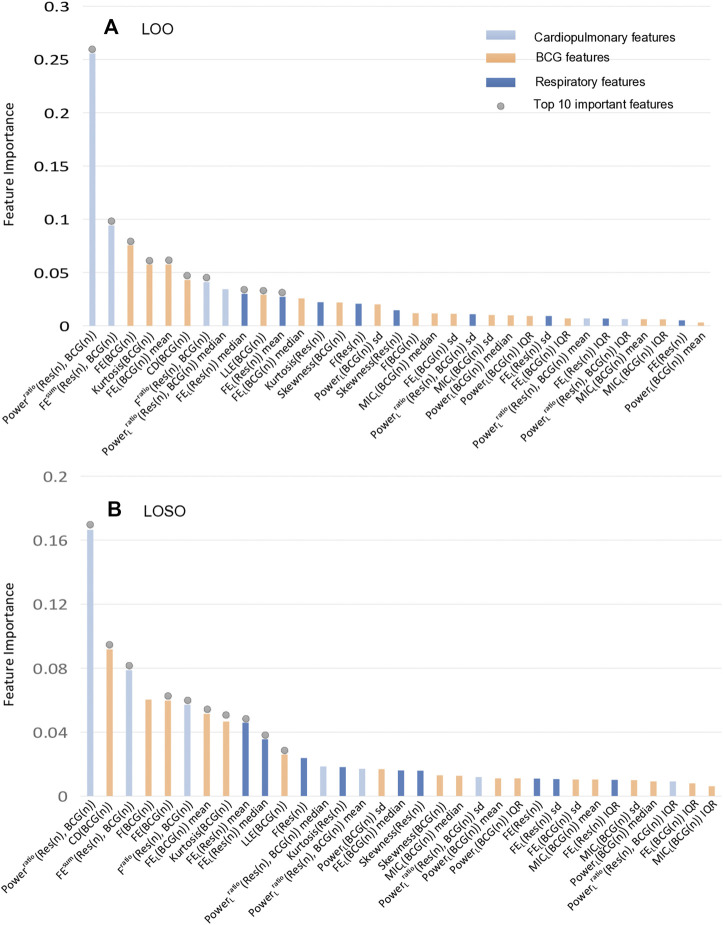
The importance ranking of all features in the XGBoost model under LOO and LOSO. The together top 10 important features contribute 71.00% and 65.88% to the classification, respectively. **(A)** The importance ranking of classifier features in LOO settings. **(B)** The importance ranking of classifier features in LOSO settings.

In the following experiments, we mainly focused on analyzing the features’ contributions to the classification/detection of the HF diseases, where the XGBoost weights were used to evaluate the features’ contributions. The details of the feature importance are shown in Figure 6 under the LOO and the LOSO settings, respectively. Specifically, the importance scores of all features were firstly obtained from the XGBoost classifier after the training phase, and then the mean value of each feature’s importance was computed. Similar to (Aydemir et al., 2019), the common top 10 features in the two experimental settings were analyzed. Among them, there are 5 features about respiratory effort (there were 2 respiratory signal independent features and 3 cardiopulmonary features). Considering the facts that there are mostly non-linear features, implying that assessing the complexity, fluctuation of BCG and respiratory effort is key to assist in the detection of HF. More importantly, *Power* ^*ratio*^ (*Res*(*n*), *BCG*(*n*)) features appear to be significantly more informative than the others, reflecting that a comprehensive assessment of individual relative power between respiratory effort and BCG signals is an important reference for the detection of HF.

## 4 Discussion

**FIGURE 7:**
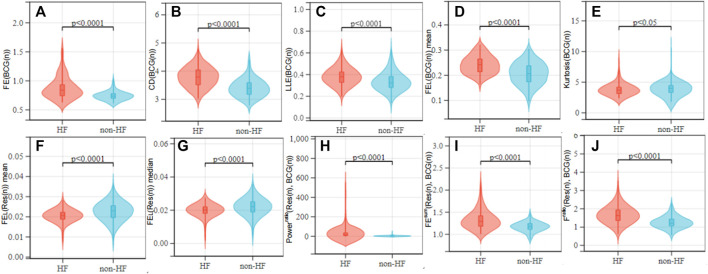
Value distribution of each feature over the HF and non-HF classes. **(A**–**E)** are BCG features; **(F**, **G)** are respiratory signal features; **(H**–**J)** are cardiopulmonary features. Non-parametric tests were used between groups.

This study proposed a non-contact piezoelectric sensing-based HF detection scheme, which can provide the robust performance for HF (LVEF ≤ 49%) detection without the quality assessment of BCG signals. Considering that the HF is the end-stage of all cardiovascular diseases, many HF patients usually have mitral and tricuspid regurgitation and suffer from low vascular compliance (Cruickshank, 2007). These kinds of heart diseases may cause the irregularity in the beat-to-beat BCG morphology, and bring challenge in the HF detection (Aydemir et al., 2019; Chang et al., 2020). To reduce the above challenge, we proposed a HF detection method, which is available to the BCG signals with different complex morphologies by using many linear and non-linear features. From the viewpoint of the BCG feature extraction, it is considered that HF patients may have the reduced amplitude, the reduced power, the significant morphology diversity and the poor regularity due to the reduced ventricular systolic function and the unstable myocardial motor performance. To better exploit the signal morphology, we also extracted the non-linear BCG features including the FE, the LLE and the CD of the chaos, and the high order statistic kurtosis. As shown in Figures 7A–E, both of the four Chaos features (*FE* (*BCG*(*n*)), *CD* (*BCG*(*n*)), *LLE* (*BCG*(*n*)), *FE*_*L*_ (*BCG*(*n*))*mean*) had the low p-values (p <0.0001 ) between the HF and the healthy cohorts, and the high order feature *Kurtosis* (*BCG*(*n*)) showed the p-values as *p* < 0.05. Therefore, by using more non-linear and high order statistic features, our proposed HF detection method is robust to the BCG signals.

On the other hand, HF patients with the lower CO usually result in the reduced gas exchange capacity of the lungs, that the human body compensates by accelerating the respiratory rate and amplitude to the regulate hypoxia. Consequently, the presence or the absence of the breath shortness is considered an important reference in the clinical diagnosis of the HF (McDonagh et al., 2021). However, there is a lack of analysis and usage of the respiratory characteristics in the existing HF detection studies related to the wearable devices. Motivated by that and considering that the HF patients are easy to show the breath shortness and the enhanced respiratory effort in the supine posture, the proposed HF detection algorithm was designed for the acquisition of sign data in the supine (soldier sleeping position) posture (Liu et al., 2015). Figures 7F, G shows that due to the HF patients have enhanced respiratory effort, respiratory signal complexity features (*FE*_*L*_ (*Res*(*n*)))*mean*, *FE*_*L*_ (*Res*(*n*)))*median*) were lower than the healthy group (p <0.0001 ). According to the relationship between the heart and the lung systems, the used features are not only associated with the BCG signals, but also related to the respiratory effort signals including many cardiopulmonary features (the relative power *Power* ^*ratio*^ (*Res*(*n*), *BCG*(*n*)), the relative amplitude *F* ^*ratio*^ (*Res*(*n*), *BCG*(*n*)) and the overall complexity *FE* ^*sum*^ (*Res*(*n*), *BCG*(*n*)))). The detailed results are shown in Figures 7H–J, where the HF and the healthy cohorts had significant difference in statistic with the low p-values (p <0.0001 ). They also demonstrated that the respiratory-related (respiratory and cardiopulmonary) features provide the key contribution and should be analyzed in the HF detection.

Compared with the existing studies of wearable sensor-aided HF detection, the proposed scheme has the advantage of automatic HF classification in a non-contact manner, and also performs feasible to the detection of HF patients with potential irregular heart rhythm (whose recorded BCG are unable to identify). It is thus expected that the proposed scheme has the potential for people with limited mobility carrying out in-home HF detection.

## 5 Conclusion

In this paper, a machine learning-based scheme was proposed for the HF detection by using the BCG, the respiratory and the cardiopulmonary features. Comparing with existing studies focusing on the BCG signals, our proposed scheme fully exploit the relationship between the heart and the lung systems. The experiment results verified that these above features can significantly improve the accuracy performance and the robustness of the HF detection. In the further step of our study, quantitative analysis for possible classification between HF patients with LVEF ≤ 40% and LVEF > 40% will be considered.

## Data Availability

The raw data supporting the conclusions of this article will be made available by the authors, without undue reservation.

## References

[B1] Ashouri H. Orlandic L. Inan O. T. (2016). Unobtrusive estimation of cardiac contractility and stroke volume changes using ballistocardiogram measurements on a high bandwidth force plate. Sensors 16, 787. 10.3390/s16060787 27240380 PMC4934213

[B2] Aydemir V. B. Nagesh S. Shandhi M. M. H. Fan J. Klein L. Etemadi M. (2019). Classification of decompensated heart failure from clinical and home ballistocardiography. IEEE Trans. Biomed. Eng. 67, 1303–1313. 10.1109/tbme.2019.2935619 31425011 PMC7271768

[B3] Bruser C. Diesel J. Zink M. D. Winter S. Schauerte P. Leonhardt S. (2012). Automatic detection of atrial fibrillation in cardiac vibration signals. IEEE J. Biomed. Health Inf. 17, 162–171. 10.1109/titb.2012.2225067 23086532

[B4] Carlson C. Turpin V. R. Suliman A. Ade C. Thompson D. E. (2020). Bed-based ballistocardiography: Dataset and ability to track cardiovascular parameters. Sensors 21, 156. 10.3390/s21010156 33383739 PMC7795624

[B5] Chang I. S. Mak S. Armanfard N. Boger J. Grace S. L. Arcelus A. (2020). Quantification of resting-state ballistocardiogram difference between clinical and non-clinical populations for ambient monitoring of heart failure. IEEE J. Transl. Eng. Health Med. 8, 2700811–11. 10.1109/jtehm.2020.3029690 33094034 PMC7571868

[B6] Chen T. Guestrin C. (2016). “Xgboost: A scalable tree boosting system,” in Proceedings of the 22nd acm sigkdd international conference on knowledge discovery and data mining, 785–794. 10.1145/2939672.2939785

[B7] Chen W. Wang Z. Xie H. Yu W. (2007). Characterization of surface EMG signal based on fuzzy entropy. IEEE Trans. Neural Syst. Rehabilitation Eng. 15, 266–272. 10.1109/tnsre.2007.897025 17601197

[B8] Cruickshank J. (2007). Are we misunderstanding beta-blockers. Int. J. Cardiol. 120, 10–27. 10.1016/j.ijcard.2007.01.069 17433471

[B9] de Vries H. Jonkman A. Shi Z.-H. Spoelstra-de Man A. Heunks L. (2018). Assessing breathing effort in mechanical ventilation: Physiology and clinical implications. Ann. Transl. Med. 6, 387–401. 10.21037/atm.2018.05.53 30460261 PMC6212364

[B10] Dickinson M. G. Allen L. A. Albert N. A. Salvo T. D. Ewald G. A. Vest A. R. (2018). Remote monitoring of patients with heart failure: A white paper from the heart failure society of America scientific statements committee. J. Cardiac Fail. 24, 682–694. 10.1016/j.cardfail.2018.08.011 30308242

[B11] Ertuğrul Ö. F. Tağluk M. E. (2017). A novel version of k nearest neighbor: Dependent nearest neighbor. Appl. Soft Comput. 55, 480–490. 10.1016/j.asoc.2017.02.020

[B12] Etemadi M. Hersek S. Tseng J. M. Rabbani N. Heller J. A. Roy S. (2014). “Tracking clinical status for heart failure patients using ballistocardiography and electrocardiography signal features,” in 2014 36th Annual International Conference of the IEEE Engineering in Medicine and Biology Society, Chicago, IL, USA, 26-30 August 2014, 5188–5191. 10.1109/embc.2014.6944794 PMC4600348 25571162

[B13] Giovangrandi L. Inan O. T. Banerjee D. Kovacs G. T. (2012). Preliminary results from BCG and ECG measurements in the heart failure clinic. Annu. Int. Conf. IEEE Eng. Med. Biol. Soc. 2012, 3780–3783. 10.1109/embc.2012.6346790 23366751

[B14] Gupta V. Mittal M. Mittal V. (2019). R-peak detection using chaos analysis in standard and real time ecg databases. Innovation Res. Biomed. Eng. 40, 341–354. 10.1016/j.irbm.2019.10.001

[B15] Hamazaki N. Masuda T. Kamiya K. Matsuzawa R. Nozaki K. Ichikawa T. (2019). 298Change in respiratory muscle strength predicts clinical events in patients with chronic heart failure. Eur. Heart J. 40, ehz747–0095. 10.1093/eurheartj/ehz747.0095

[B16] Hao G. Wang X. Chen Z. Zhang L. Zhang Y. Wei B. (2019). Prevalence of heart failure and left ventricular dysfunction in China: The China hypertension survey, 2012–2015. Eur. J. Heart Fail. 21, 1329–1337. 10.1002/ejhf.1629 31746111

[B17] Inan O. T. Etemadi M. Paloma A. Giovangrandi L. Kovacs G. (2009). Non-invasive cardiac output trending during exercise recovery on a bathroom-scale-based ballistocardiograph. Physiol. Meas. 30, 261–274. 10.1088/0967-3334/30/3/003 19202234

[B18] Jingxian L. Jialin H. Liwei M. Baoxian Y. Pengbin C. Zhiqiang P. (2020). An effective algorithm for beat-to-beat heart rate monitoring from ballistocardiograms. J. Med. Imaging Health Inf. 10, 633–640. 10.1166/jmihi.2020.2910

[B19] Li M. Wang R. Xu D. (2020). An improved composite multiscale fuzzy entropy for feature extraction of MI-EEG. Entropy 22, 1356. 10.3390/e22121356 33266204 PMC7761434

[B20] Liu J. Miao F. Yin L. Pang Z. Li Y. (2021). A noncontact ballistocardiography-based IoMT system for cardiopulmonary health monitoring of discharged COVID-19 patients. IEEE Internet Things J. 8, 15807–15817. 10.1109/jiot.2021.3063549 35782189 PMC8768984

[B21] Liu X. Cao J. Tang S. Wen J. Guo P. (2015). Contactless respiration monitoring via off-the-shelf wifi devices. IEEE Trans. Mob. Comput. 15, 2466–2479. 10.1109/tmc.2015.2504935

[B22] Magrelli S. Valentini P. De Rose C. Morello R. Buonsenso D. (2021). Classification of lung disease in children by using lung ultrasound images and deep convolutional neural network. Front. Physiology 12, 693448. 10.3389/fphys.2021.693448 PMC8432935 34512375

[B23] Mai Y. Chen Z. Yu B. Li Y. Pang Z. Han Z. (2022). Non-contact heartbeat detection based on ballistocardiogram using UNet and bidirectional long short-term memory. IEEE J. Biomed. Health Inf. 26, 3720–3730. 10.1109/jbhi.2022.3162396 35333727

[B24] McDonagh T. A. Metra M. Adamo M. Gardner R. S. Baumbach A. Böhm M. (2021). 2021 ESC guidelines for the diagnosis and treatment of acute and chronic heart failure: Developed by the task force for the diagnosis and treatment of acute and chronic heart failure of the European society of cardiology (ESC) with the special contribution of the heart failure association (HFA) of the ESC. Eur. Heart J. 42, 3599–3726. 10.1093/eurheartj/ehab368 34649282

[B25] Mozziyar E. T Omer I. Laurent G. Ta Gregory K. (2011). Rapid assessment of cardiac contractility on a home bathroom scale. IEEE Trans. Inf. Technol. Biomed. 15, 864–869. 10.1109/titb.2011.2161998 21843998

[B26] Palaniappan R. Sundaraj K. Sundaraj S. (2014). A comparative study of the SVM and K-nn machine learning algorithms for the diagnosis of respiratory pathologies using pulmonary acoustic signals. BMC Bioinforma. 15, 223. 10.1186/1471-2105-15-223 PMC4094993 24970564

[B27] Park J. J. Park J.-B. Park J.-H. Cho G.-Y. (2018). Global longitudinal strain to predict mortality in patients with acute heart failure. J. Am. Coll. Cardiol. 71, 1947–1957. 10.1016/j.jacc.2018.02.064 29724346

[B28] Pieske B. Tschöpe C. De Boer R. A. Fraser A. G. Anker S. D. Donal E. (2019). How to diagnose heart failure with preserved ejection fraction: The HFA–PEFF diagnostic algorithm: A consensus recommendation from the heart failure association (HFA) of the European society of cardiology (ESC). Eur. heart J. 40, 3297–3317. 10.1093/eurheartj/ehz641 31504452

[B29] Procacia I. Procaccia I. (1983). Measuring the strangeness of strange attractors. Phys. D. 9, 189–208. 10.1016/0167-2789(83)90298-1

[B30] Reshef D. N. Reshef Y. A. Finucane H. K. Grossman S. R. McVean G. Turnbaugh P. J. (2011). Detecting novel associations in large data sets. Science 334, 1518–1524. 10.1126/science.1205438 22174245 PMC3325791

[B31] Rosenstein M. T. Collins J. J. De Luca C. J. (1993). A practical method for calculating largest Lyapunov exponents from small data sets. Phys. D. Nonlinear Phenom. 65, 117–134. 10.1016/0167-2789(93)90009-p

[B32] Savarese G. Becher P. M. Lund L. H. Seferovic P. Rosano G. Coats A. J. (2022). Global burden of heart failure: A comprehensive and updated review of epidemiology. Cardiovasc. Res. 00, 1–16. 10.1093/cvr/cvac013 35150240

[B33] Shi Y. Yao X. Xu J. Hu X. Tu L. Lan F. (2022). A new approach of fatigue classification based on data of tongue and pulse with machine learning. Front. Physiology 12, 708742–712146. 10.3389/fphys.2021.708742 PMC8859319 35197858

[B34] Siniorakis E. Arvanitakis S. Tsitsimpikou C. Tsarouhas K. Limberi S. Panta S. (2018). Acute heart failure in the emergency department: Respiratory rate as a risk predictor. Vivo 32, 921–925. 10.21873/invivo.11330 PMC6117786 29936481

[B35] Society of Cardiology C. (2018). Chinese guidelines for the diagnosis and treatment of heart failure 2018. Chin. J. Cardiovasc. Dis. 46, 760–789. 10.3760/cma.j.issn.0253-3758.2018.10.004 30369168

[B36] Starr I. Rawson A. J. Schroeder H. A. Joseph N. R. (1939). Studies on the estimation of cardiac output in man, and of abnormalities in cardiac function, from the heart’s recoil and the blood’s impacts; the ballistocardiogram. Am. J. Physiology 127, 1–28. 10.1152/ajplegacy.1939.127.1.1

[B37] Starr I. Schroeder H. A. (1940). Ballistocardiogram. II. normal standards, abnormalities commonly found in diseases of the heart and circulation, and their significance. J. Clin. Investigation 19, 437–450. 10.1172/jci101145 PMC434977 16694759

[B38] Sun J. Yu H. Zhong G. Dong J. Zhang S. Yu H. (2020). Random shapley forests: Cooperative game-based random forests with consistency. IEEE Trans. Cybern. 52, 205–214. 10.1109/tcyb.2020.2972956 32203041

[B39] Wen X. Yanqi H. Xiaomei W. Biyong Z. (2019). A feasible feature extraction method for atrial fibrillation detection from BCG. IEEE J. Biomed. Health Inf. 24, 1093–1103. 10.1109/jbhi.2019.2927165 31295128

